# The mitochondrial genome of *Apis mellifera simensis* (Hymenoptera: Apidae), an Ethiopian honey bee

**DOI:** 10.1080/23802359.2019.1693307

**Published:** 2019-12-10

**Authors:** Leigh Boardman, Amin Eimanifar, Rebecca Kimball, Edward Braun, Stefan Fuchs, Bernd Grünewald, James D. Ellis

**Affiliations:** a Honey Bee Research and Extension Laboratory, Entomology and Nematology Department, University of Florida, Gainesville, FL, USA;; b Independent Senior Research Scientist, Industrial District, Easton, MD, USA;; c Department of Biology, University of Florida, Gainesville, FL, USA;; d Institut für Bienenkunde, Goethe-Universität Frankfurt am Main, Oberursel, Germany

**Keywords:** Mitogenome, next-generation sequencing, A-lineage honey bee, Ethiopian honey bee

## Abstract

The complete mitochondrial genome of *Apis mellifera simensis* was 16,523 bp long. The 13 protein-coding genes, two rRNAs, and 22 tRNAs resembled other *Apis* mitogenomes. The location of this *Apis* subspecies in our phylogenetic tree supported the hypothesis that this subspecies is distinct, and is most closely related to *A. m. scutellata* and *A. m. monticola*.

*Apis mellifera simensis* is found in the volcanic dome system in Ethiopia. First described by Meixner et al. (2011) using morphometrics, this subspecies appears to be distinct from the other African honey bees found in the surrounding areas, *A. m. monticola*, *A. m. scutellata*, *A. m. jemenitica*, and *A. m. litorea*. Despite the interest in studying honey bee biodiversity of north-eastern Africa, the genetics of *A. m. simensis* have not been investigated. Here, we present the complete mitochondrial genome of a worker honey bee from the Ruttner Bee Collection at the Bee Research Institute in Oberursel, Germany (Voucher No. 2721, Messele Abebe, 1998, Gimbi, Ethiopia, 9°10N, 35°50E). The identification was confirmed as *A. m. simensis* using morphometrics. GenBank accession number MN585108.

Total genomic DNA was extracted and sequenced using next-generation sequencing technology (PE-150bp, Illumina Hi-Seq 3000/4000, San Diego, CA, USA, details in Eimanifar et al. (2017)). Quality control was performed using FastQC (Andrews 2010) before reads were trimmed with Trimmomatic (Bolger et al. 2014). Reads were mapped to eight *A. mellifera* mitogenomes in Geneious Prime 2019.0.4 (Biomatters Ltd., Auckland, New Zealand) (Kearse et al. 2012) following Boardman et al. (2019) with *A. m. sahariensis* (MF351881) having the highest pairwise identity. As this approach resulted in many ambiguities, we mapped the R1 paired trimmomatic output to *A. m. sahariensis* with increased stringency: 10 iterations and 95% minimum overlap identity. The resulting mitogenome was annotated with mitos2 (Bernt et al. 2013) and manually adjusted to the *A. m. capensis* (KX870183) annotation in Geneious. The phylogenetic tree (Figure 1) was estimated by manually aligning the 13 protein-coding genes (PCGs) and two ribosomal RNAs (rRNAs) in Mesquite version 3.5 (Maddison and Maddison 2018), running RAxML 8.2.10 (GTRGAMMA, 1000 bootstrap replicates, -f a option)(Stamatakis 2014) on CIPRES Science Gateway version 3.3 (Miller et al. 2010). The *P*-distances were obtained using PAUP 4.0a (Swofford 2003).

**Figure 1.**
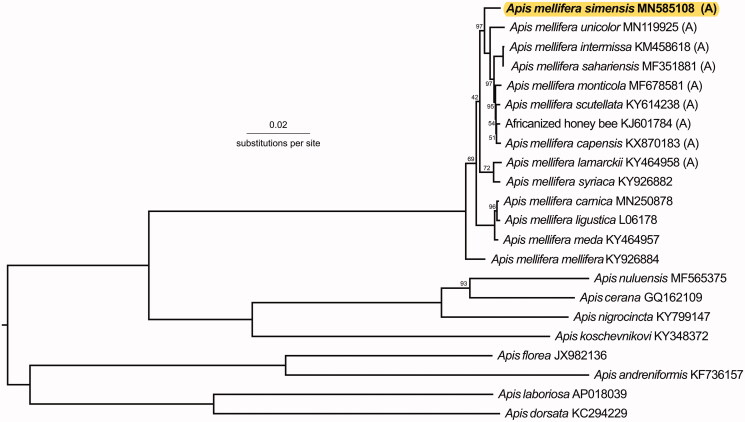
A phylogenetic tree showing the relationship between *A. m. simensis* (GenBank: MN585108) and 21 other *Apis* honey bees (GenBank accession numbers provided), with (A) indicating honey bees from the African A-lineage. Node labels indicate the bootstrap values and unlabeled lineages are 100%. The tree is midpoint rooted.

The complete mitogenome was 16,523 bp and consisted of 43.1% A, 41.7% T, 9.6% C, and 5.6% G. The location of the 13 PCGs was identical to that for other *Apis* mitogenomes with four PCGs on the heavy strand, and nine on the light strand, with 19 shared nucleotides between *atp8* and *atp6.* Similarly, ATT was the start codon for six PCGs, ATG for four, ATA for two, and ATC for one; while all 13 ended with TAA. The mitogenome consisted of 22 transfer RNAs (tRNAs) and two rRNAs. The shortest tRNA was tRNA-Gln (62 bp) and the longest was tRNA-Thr (79 bp). Both rRNAs were AT rich with >80% AT and were located on the heavy strand. The 16S rRNA was 1325 bp and the 12S rRNA was 785 bp.

Our phylogeny (Figure 1) supported the assertion that *A. m. simensis* is indeed a unique subspecies. It was most closely related to *A. m. scutellata* (*P*-distance: 0.00854) and *A. m. monticola* (*P*-distance: 0.00898). Additional research on the mitogenomes of other east African subspecies *A. m. litorea* and *A. m. jemenitica* should reveal insights into the honey bees of this area.
